# Cisplatin, gemcitabine, and treosulfan in relapsed stage IV cutaneous malignant melanoma patients

**DOI:** 10.1038/sj.bjc.6604045

**Published:** 2007-10-30

**Authors:** J Atzpodien, K Terfloth, M Fluck, M Reitz

**Affiliations:** 1 Fachklinik Hornheide an der Westfälischen Wilhelms-Universität Münster, Dorbaumstr. 300, Münster 48157, Germany; 2 Europäisches Institut für Tumor Immunologie und Prävention (EUTIP), Bad Honnef, Germany

**Keywords:** melanoma, cisplatin, gemcitabine, treosulfan

## Abstract

To evaluate the efficacy of cisplatin, gemcitabine, and treosulfan (CGT) in 91 patients with pretreated relapsed AJCC stage IV cutaneous malignant melanoma. Patients in relapse after first-, second-, or third-line therapy received 40 mg m^−2^ intravenous (i.v.) cisplatin, 1000 mg m^−2^ i.v. gemcitabine, and 2500 mg m^−2^ i.v. treosulfan on days 1 and 8. Cisplatin, gemcitabine, and treosulfan therapy was repeated every 5 weeks until progression of disease occurred. A maximum of 11 CGT cycles (mean, two cycles) was administered per patient. Four patients (4%) showed a partial response; 15 (17%) patients had stable disease; and 72 (79%) patients progressed upon first re-evaluation. Overall survival of all 91 patients was 6 months (2-year survival rate, 7%). Patients with partial remission or stable disease exhibited a median overall survival of 11 months (2-year survival rate, 36%), while patients with disease progression upon first re-evaluation had a median overall survival of 5 months (2-year survival rate, 0%). Treatment with CGT was efficient in one-fifth of the pretreated relapsed stage IV melanoma patients achieving disease stabilisation or partial remission with prolonged but limited survival.

The prognosis for malignant melanoma patients with distant metastases is poor. Even though a small proportion of patients can attain long-term survival with systemic therapy, the median survival of advanced melanoma patients is about 6 months. While interferon-*α* continues to be standard in the adjuvant therapy of resected high-risk melanoma (Hillner *et al*, 1997), dacarbazine (DTIC) has been the most widely used agent in the first-line treatment of stage IV metastatic melanoma, yielding a response rate of up to 20% (Serrone *et al*, 2000). Although several DTIC-based chemotherapy and chemoimmunotherapy combinations have been reported with response rates between 34 and 53% (Richards *et al*, 1992; Huncharek *et al*, 2001; Atzpodien *et al*, 2002; Stein *et al*, 2002), these regimens have not yielded a significant survival advantage. Once DTIC-based therapy has failed, no standard systemic treatment has been available for relapsed IV-stage malignant melanoma patients.

Preclinical studies on the chemosensitivity of metastatic melanoma cells to cytotoxic agents identified sensitivity while using combinations of gemcitabine with treosulfan and gemcitabine with cisplatin (Cree *et al*, 1999; Ugurel *et al*, 2003). First results of a phase II trial of 24 metastatic uveal melanoma patients treated with gemcitabine and treosulfan showed a prolonged progression-free survival and a slight increase in tumour responses, when compared to 24 patients treated with treosulfan alone (Schmittel *et al*, 2006).

The goal of our present analyses was to evaluate the efficacy of combined cisplatin, gemcitabine, and treosulfan in pretreated relapsed stage IV malignant melanoma patients.

## PATIENTS AND METHODS

### Patients

Between February 2001 and August 2006, 91 relapsed stage IV cutaneous melanoma patients received a combination treatment with cisplatin, gemcitabine, and treosulfan (CGT). At start of CGT therapy, patients showed one metastatic site (*n*=46), two metastatic sites (*n*=29), three metastatic sites (*n*=13), and four metastatic sites (*n*=2), respectively. Pretreatment serum lactate dehydrogenase level was elevated in 51% of the patients.

Criteria for entry into the study were: systemically pretreated relapsed AJCC stage IV cutaneous malignant melanoma; white blood cell count >3500 *μ*l^−1^; platelet count >100 000 *μ*l^−1^; haematocrit >30%; serum creatinin and bilirubin <1.5 of the upper normal limit; age between 18 and 80 years, and a life expectancy of >3 months. Progressive CNS metastases were no exclusion criteria. Previous systemic cisplatin failures were not excluded, since cisplatin was used in combination only.

All patients had a Karnofsky performance status >80%.

Written informed consent was obtained from all patients prior to therapy.

### Treatment design

Patients in relapse after first-, second-, or third-line therapy received 40 mg m^−2^ intravenous (i.v.) cisplatin, 1000 mg m^−2^ i.v. gemcitabine, and 2500 mg m^−2^ i.v. treosulfan on days 1 and 8. Therapy was repeated every 5 weeks until progression of disease occurred.

Dose and schedule was employed according to Pfohler *et al* (2003) and Schmittel *et al* (2005). Cisplatin was added at a moderate standard dose. Fifty-nine (65%) patients required a dose reduction due to toxicity.

### Response, survival, and toxicity

Response to therapy was evaluated according to World Health Organization (WHO) criteria with regular re-evaluation intervals every 2 months; complete response: disappearance of all signs of disease for a minimum of 2 months; partial response: 50% or more reduction in the sum of products of the greatest perpendicular diameters of measurable lesions, no increase in lesion size, and no new lesions; stable disease: less than a partial response with no disease progression for at least 5 weeks; progressive disease: 25% or more increase in sum of products of the longest perpendicular diameters of measurable lesions or the development of new lesions.

Survival was measured from start of therapy to date of death or to the last known date to be alive.

Maximum toxicity was evaluated according to WHO criteria.

### Statistical analysis

The statistical end points in our analysis were: (1) rate of response or disease stabilisation (primary end point) and (2) overall survival of patients.

The response rate (SD, PR) for patients in relapse after previous systemic chemotherapy was hypothesised to be at least 5%. Using an *α* of 0.05 (two-sided), a sample size of 73 patients was needed to have 80% power to statistically establish the assumed response rate. Given the tumour-related patient morbidity, up to 25% drop-out rate was estimated.

The probability of overall survival and progression-free survival was plotted over time according to the method of Kaplan and Meier; SPSS software (SPSS Inc., Chicago, IL, USA) was employed.

## RESULTS

Median follow-up of all patients was 6 months (range: 0–29 months). Patient characteristics are listed in Table 1. The patient group consisted of 59 men and 32 women, at a median age of 58 years. Seventy-two patients had a cutaneous primary, while in 19 patients, the primary was unknown. All patients had failed previous therapy. Stage IV pretreatment consisted of chemotherapy, notably, DTIC, Cisplatin, BCNU/Fotemustine (*n*=68); DTIC, BCNU, Hydroxyurea (*n*=23); DTIC, Cisplatin+Vinblastine (*n*=4); DTIC+Roferon (*n*=4); BCNU/Fotemustine+Bleomycin, Vindesine (*n*=3); Trofosfamide+Treosulfan, Gemcitabine (*n*=3); and Temozolamide (*n*=2). Patients received a mean of two CGT cycles (range: 1–11) until progression of disease occurred or until last known date to be alive.

### Outcome

Four patients (4%) reached a partial remission, 15 (17%) patients had stable disease, and 72 (79%) patients exhibited progressive disease upon first re-evaluation (Table 1).

There was no significant difference in treatment response between cisplatin-pretreated patients (6% PR; 17% SD; 77% PD) and noncisplatin-pretreated patients (13% SD; 87% PD).

At the last follow-up, 3 (3%) of the 91 patients are progression-free (range: 5–26 months). Six-month and 12-month progression-free survival was calculated at 8.7 and 5.8%, respectively.

### Survival

**Figure 1:**
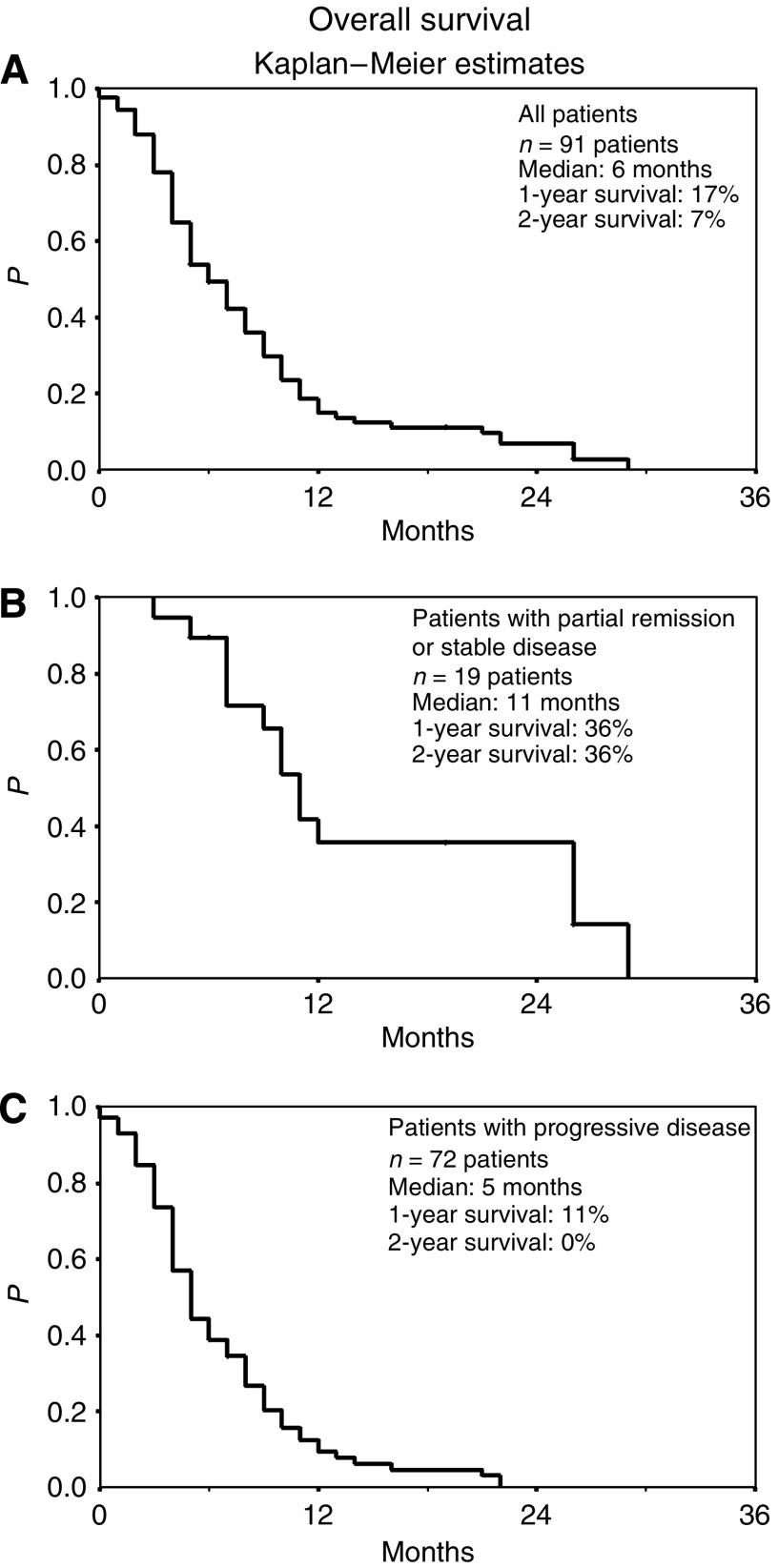
Overall survival (Kaplan–Meier estimates) of (**A**) all 91 patients, (**B**) 19 patients with partial remission or stable disease, and (**C**) 72 patients with progressive disease. Patients were treated with cisplatin, gemcitabine, and treosulfan. Survival was measured from start of therapy.

Overall media survival of all 91 patients was 6 months (range: 0–29 months; 1-year survival rate, 17%, 2-year survival rate, 7%) (Figure 1A). Patients achieving a partial remission or stable disease (*n*=19) exhibited a median overall survival of 11 months (1 and 2-year survival rate, 36%) (Figure 1), while patients with disease progression (*n*=72) upon first evaluation showed a median overall survival of 5 months (1-year survival rate, 11%; 2-year survival rate, 0%) (Figure 1). At the last follow-up, 7 (8%) patients (range: 5–26 months) are alive.

### Treatment toxicity

Cisplatin, gemcitabine, and treosulfan therapy was moderate-to-well tolerated. No toxic deaths occurred. Most haematologic side effects were limited to WHO grades I and II and were experienced in 52% (leukopenia), 50% (anaemia), and 29% (thrombocytopenia) of CGT-treated patients; grade III- or IV-related haematologic side effects were experienced in 26% (leukopenia), 20% (thrombocytopenia), and 3% (anaemia) of patients (Table 2). Eighty-seven per cent of patients showed no major (WHO grade III/IV) nausea/vomiting, 98% of patients experienced no major (WHO grade III/IV) polyneuropathy, and 78% of patients showed no other significant toxicities according to WHO (grade III/IV).

## DISCUSSION

The prognosis of patients with relapsed high-risk melanoma failing standard DTIC-based chemotherapy remains disappointing. However, recent preclinical studies on the chemosensitivity of metastatic melanoma cells to cytotoxic agents identified high sensitivity to cytotoxic single agents including cisplatin, treosulfan, gemcitabine, as well as to combinations of gemcitabine plus treosulfan and gemcitabine plus cisplatin (Cree *et al*, 1999; Ugurel *et al*, 2003, 2006).

Our present analysis of 91 high-risk AJCC stage IV melanoma patients failing previous first-, second-, or third-line therapy, showed a median overall survival of 6 months upon subsequent treatment with the combination of CGT.

While this is the first report on the effect of CGT on metastatic cutaneous melanoma, recent results on CGT-treated metastatic uveal melanoma patients showed a similar median overall survival of 7.7 months (Schmittel *et al*, 2005). Other authors reported that 14 metastatic uveal melanoma patients treated with gemcitabine and treosulfan as first-line therapy (except one) yielded an objective reponse of 28% and a median overall survival of 61 weeks (Pfohler *et al*, 2003).

This present multi-agent chemotherapy regimen led to 21% of patients with stable disease or partial remission, with a prolonged median overall survival of 11 months. This was even more striking given the number of prior therapies and the high percentage of cisplatin-pretreated patients.

However, there was no difference in response between cisplatin-pretreated and noncisplatin-pretreated patients suggesting that relapse after previous systemic therapy, that is, prior to the current regimen, may have been cisplatin unrelated.

In the present group of pretreated metastatic cutaneous melanoma patients, median overall survival upon CGT treatment was similar to that reported upon standard DTIC-based therapy, which yielded a median overall survival of 7 months (Chapman *et al*, 1999). Similar historical outcome upon current CGT second-/third-/fourth-line therapy compared with DTIC-based first-line treatment might be explained by treatment eligibility-related patient selection; thus, only patients with Karnofsky performance status >80% despite relapse of disease received subsequent CGT therapy, here.

In summary, treatment with CGT was efficient in one-fifth of pretreated relapsed stage IV melanoma patients achieving disease stabilisation or partial remission. In future, it could be beneficial to prospectively identify melanoma patients, who could benefit from selected chemotherapeutic agents after failing first- or second-line standard chemotherapy.

## Figures and Tables

**Table 1:** Patient characteristics

	**CGT**
Entered	91
	
*Age* (*years*)	
Median	58
Range	18–80
	
*Sex*	
Male	59
Female	32
	
*Primary*	
Cutaneous	72
Unknown	19
	
*CGT*	
Second line	77
Third line	12
Fourth line	2
	
*Stage IV pretreatment* a	
DTIC, Cisplatin, BCNU/Fotemustine	68
DTIC, BCNU, Hydroxyurea	23
DTIC,Cisplatin±Vinblastine	4
DTIC±Roferon	4
BCNU/Fotemustine±Bleomycin, Vindesine	3
Trofosfamide±Treosulfan, Gemcitabine	3
Temozolamide	2
	
*Sites of progressive metastatic disease*	
Skin/soft tissue	38
Lung	33
Visceral	33
Lymph nodes	20
Bone	11
CNS	4
Others	2
	
*Maximum response*	
Complete remission	0
Partial remission	4
Stable disease	15
Progressive disease	72

Abbreviations: BCNU=carmustine; CGT=cisplatin, gemcitabine, and treosulfan; DTIC=dacarbazine

a Patients may have had more than one pre-treatment.

**Table 2:** Haematologic toxicity

**Haematologic toxicity^a^**	**% Patients**	
**WHO criteria**	**I/II**	**III/IV**
Leukocyte counts	52	26
Thrombocyte counts	29	20
Haemoglobin levels	50	3

a No life-threatening complications and no toxic deaths occurred.
